# Technological Analysis of the World’s Earliest Shamanic Costume: A Multi-Scalar, Experimental Study of a Red Deer Headdress from the Early Holocene Site of Star Carr, North Yorkshire, UK

**DOI:** 10.1371/journal.pone.0152136

**Published:** 2016-04-13

**Authors:** Aimée Little, Benjamin Elliott, Chantal Conneller, Diederik Pomstra, Adrian A. Evans, Laura C. Fitton, Andrew Holland, Robert Davis, Rachel Kershaw, Sonia O’Connor, Terry O’Connor, Thomas Sparrow, Andrew S. Wilson, Peter Jordan, Matthew J. Collins, André Carlo Colonese, Oliver E. Craig, Rebecca Knight, Alexandre J. A. Lucquin, Barry Taylor, Nicky Milner

**Affiliations:** 1 BioArCh, Department of Archaeology, University of York, York, United Kingdom; 2 School of Arts, Languages and Cultures, University of Manchester, Manchester, United Kingdom; 3 Faculty of Archaeology, Leiden University, Leiden, the Netherlands; 4 School of Archaeological Sciences, University of Bradford, Bradford, United Kingdom; 5 Arctic centre, University of Groningen, Groningen, the Netherlands; 6 Department of History and Archaeology, Department, Chester, United Kingdom; 7 Hull York Medical School, York, United Kingdom; University of Oxford, UNITED KINGDOM

## Abstract

Shamanic belief systems represent the first form of religious practice visible within the global archaeological record. Here we report on the earliest known evidence of shamanic costume: modified red deer crania headdresses from the Early Holocene site of Star Carr (c. 11 kya). More than 90% of the examples from prehistoric Europe come from this one site, establishing it as a place of outstanding shamanistic/cosmological significance. Our work, involving a programme of experimental replication, analysis of macroscopic traces, organic residue analysis and 3D image acquisition, metrology and visualisation, represents the first attempt to understand the manufacturing processes used to create these artefacts. The results produced were unexpected—rather than being carefully crafted objects, elements of their production can only be described as expedient.

## Introduction

Shamanic belief systems, the earliest form of religious practice, emerged during the Upper Palaeolithic and Mesolithic periods [1–3] and are still common today amongst hunter-gatherer and small-scale agricultural communities [4–6]. Archaeologically, shamanism is typically identified via rock art and specific types of burial practices; rich burials suggest that shamans enjoyed a high status amongst these groups, similar to that of social leaders [1, 2]. While shamanic burials have been identified from the mid-Upper Palaeolithic onwards [2, 3], none of the burials that predate the Mesolithic have preserved evidence of any form of shamanic costume. The one mooted exception to this is the unusual burial of Brno II (c. 28 kya) [3], where stone and bone roundels found with the body have been compared to discs worn by Siberian shamans. This, however, is problematic: only one of the 14 roundels is perforated, so these are unlikely to have been worn. Furthermore the burial was discovered by workmen in 1891, and the excavations lack the levels of recording required to establish a stratigraphic relationship between the artefacts and the burial itself. As a result we have little understanding of early ritual costumes, which in accounts of more recent shamanic practices seem to have played a key role in shamanic power.

Antler headdresses are an element of shamanic dress in Siberian reindeer cultures and feature in iconography from the Pleistocene, where they have also been linked to shamanic practices. At Star Carr, North Yorkshire, UK, a total of 24 red deer headdresses have been found. These headdresses date to c.11 kyr, representing c. 90% of all such known artefacts across early prehistoric Europe. Located on the edge of a paleo-lake, with good preservation of organic remains, Star Carr is one of the best known sites in Europe, and has become synonymous with our understanding of Early Mesolithic lifeways. The headdresses recovered from the site are formed from the upper part of a male red deer skull with the antlers attached—the lower jaw and cranial bones having been removed and the parietal (and occasionally frontal) bones perforated. The perforations and traces of smoothing observed on the anterior of the parietal are taken as indication of their use as headdresses [4]. In this paper we report, for the first time, detailed analysis of the method of manufacture of these earliest shamanic costumes.

Initial interpretations of the function of the Star Carr headdresses were split between use as deer disguises for hunting and shamanic costumes [5]. The former has been supported through the use of ethnographic analogies with North American groups [6–11]. However, in more recent years archaeologists have highlighted the potential for historical continuity between the inhabitants of Mesolithic Northern Europe and the more recent hunter-gatherer and pastoral groups of circumpolar Eurasia [12, 13]. Evidence for the use of deer hunting disguises within these historically documented groups is conspicuous in its absence, whilst examples of shamanic costumes featuring antlers are numerous [14]. As such, more recent discussions of the Star Carr headdresses have stressed their cosmological significance, their general role as ritualised headgear, and the lack of distinction between these supposedly separate functions within analogous ethnographic groups [15–19]. In fact, since their discovery in the 1940’s, the headdresses (along with key depictions from Upper Palaeolithic art, such as an antlered individual in the cave of Les Trois Freres, Arierge, France) have been widely used as the basis for accounts of the origin of shamanic belief systems in the European Upper Palaeolithic and Mesolithic [20,21,22].

**Fig 1:**
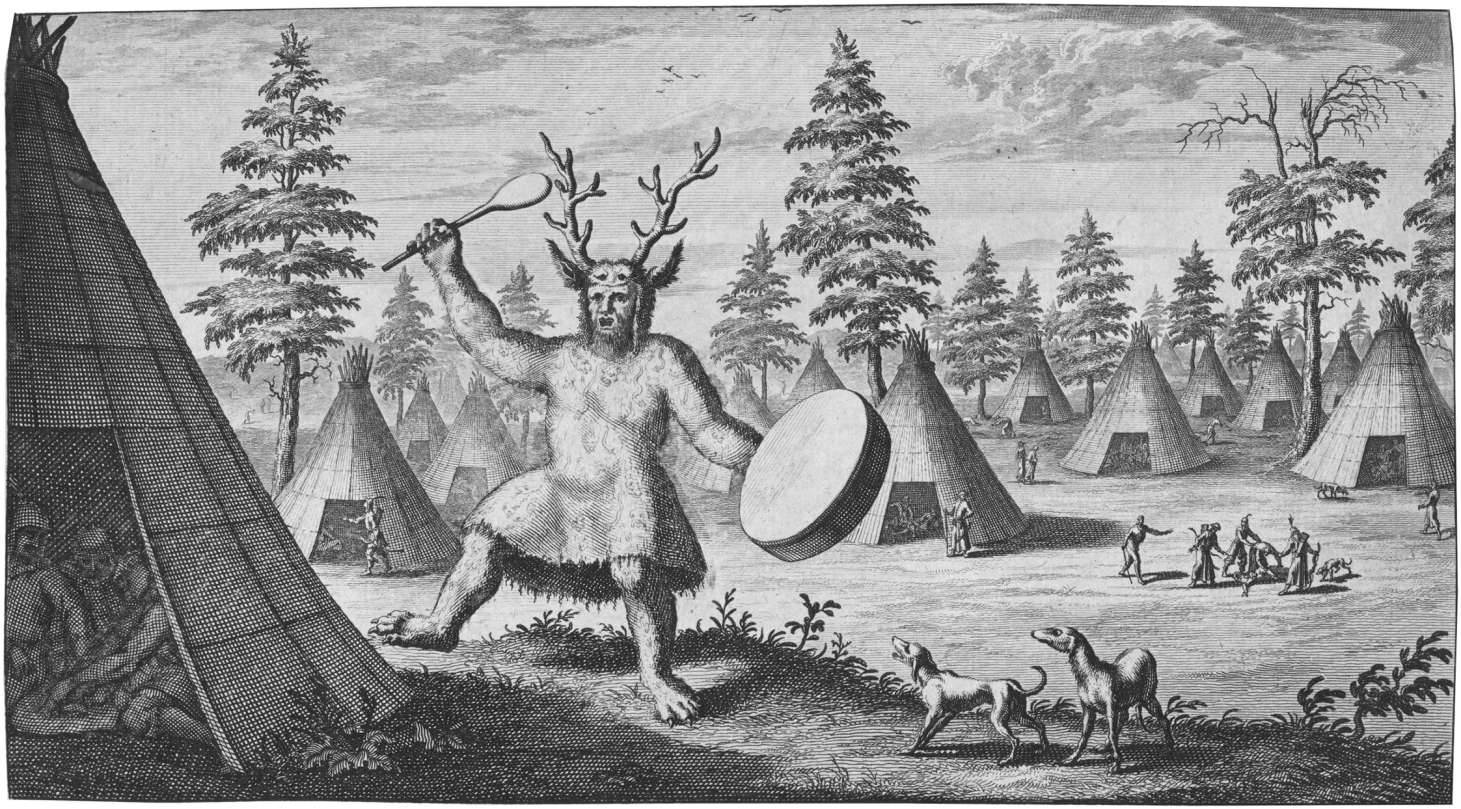
Depiction of an Evenki shaman wearing antler headdress (after Witsen 1785, 655).

Due to their rarity and socio-religious significance, in particular their connection to shamanic practices, the Star Carr headdresses have captured the imagination of scholars since their initial discovery nearly seventy years ago [23]. Shamans can be defined as religious specialists and therapists, who mediate cosmological, social and political discourses both within and between oral cultures [24]. Ethnohistorical accounts describe how shamans enter a trance state to communicate with animal spirits, often experienced as a physical transformation into the animal in question [16, 25]. During ritual ceremonies this typically involves the wearing of a costume that integrates animal references and identifies the shaman with their animal spirit [26, 27]. Costumes vary, but the wearing of a symbolic headdress representing the head of the shaman’s animal spirit is not uncommon. In the case of the South Siberian (Eveny) and Mongolian (Evenki) peoples, a deer skull cap with antlers was worn, closely resembling that of the Star Carr headdresses (Fig 1). In other instances the skins, skulls and bones are retained by, for example, the Khanty (see Fig 5.11 in [24]) or reapplied; the latter sometimes involving a ‘collaging’ of various animal pelts [28].

Recent excavations at Star Carr in 2013 uncovered three new headdresses. The most complete (find no. 103625) is represented by the frontal and parietal bones of a male *Cervus elaphus* (red deer), with pedicles and associated antler beams and tines intact. Although lacking dentition, the size and robustness of the skull combined with the development of the antlers indicates that this was a mature adult. Comparison of this specimen with examples of 20th century deer skulls from Scotland confirmed this animal to be at least 50% larger than modern counterparts.

## Methods and Materials

All animal materials used in this research were commercially available and the research conducted was approved by the Arts and Humanities Ethics Committee, University of York. The author visible in Figure 6 has given written, informed consent (as outlined in PLOS consent form) to publish these case details.

### Macroscopic analysis

**Fig 2:**
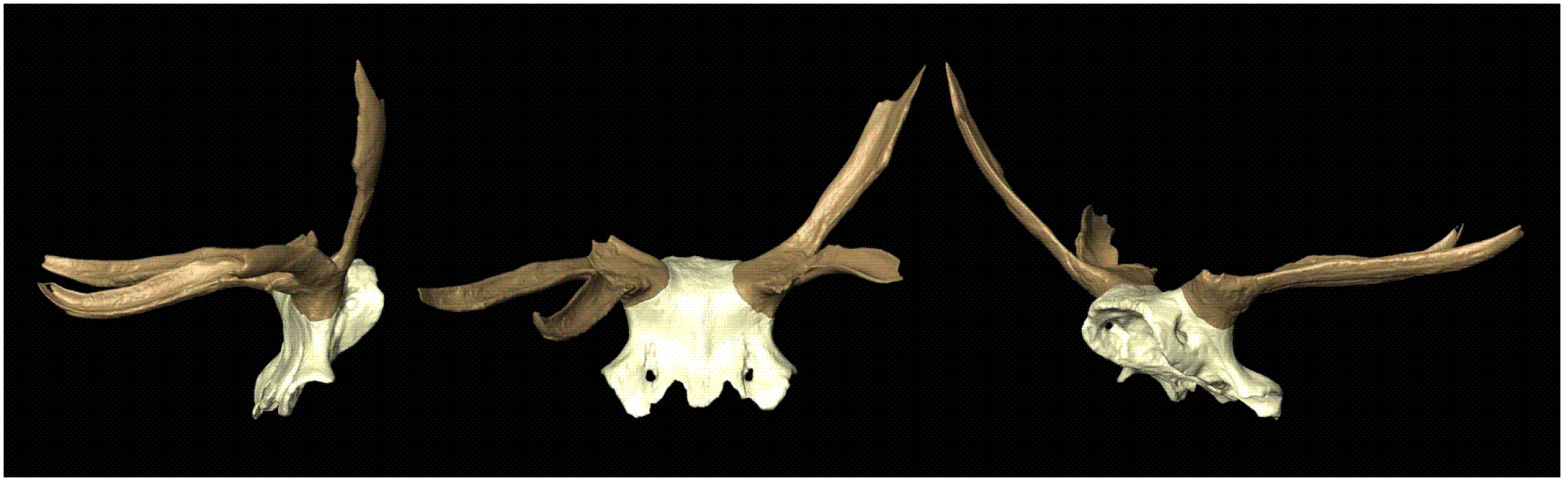
Virtual reconstruction of frontlet based on surface scans. Lateral, frontal and posterior oblique views respectively. The frontlet was reconstructed and visualised using imaging processing software Avizo 7.0–8.0 (Visualization Science Group Inc).

Due to the waterlogged conditions at Star Carr, the headdress has been stored in a wet state prior to long-term conservation. During macroscopic analysis of the headdress we observed cut marks on the interior and exterior of the crania and a number of incision-like marks radiating out of the perforations on both sides of the crania. To investigate these traces further, 3D laser scanning, structured light scanning and z-stacked macrophotography was employed. The combined use of these different approaches allowed us to overcome analytical limitations produced by the reflectivity caused by its waterlogged state. These methods also enabled accurate digital documentation of the artefact in advance of conservation. Complementary digitisation tools revealed fine surface detail, whilst the production of high fidelity 3D models of the various antler fragments allowed it to be digitally refitted, making the object ‘complete’ (Fig 2).

**Fig 3:**
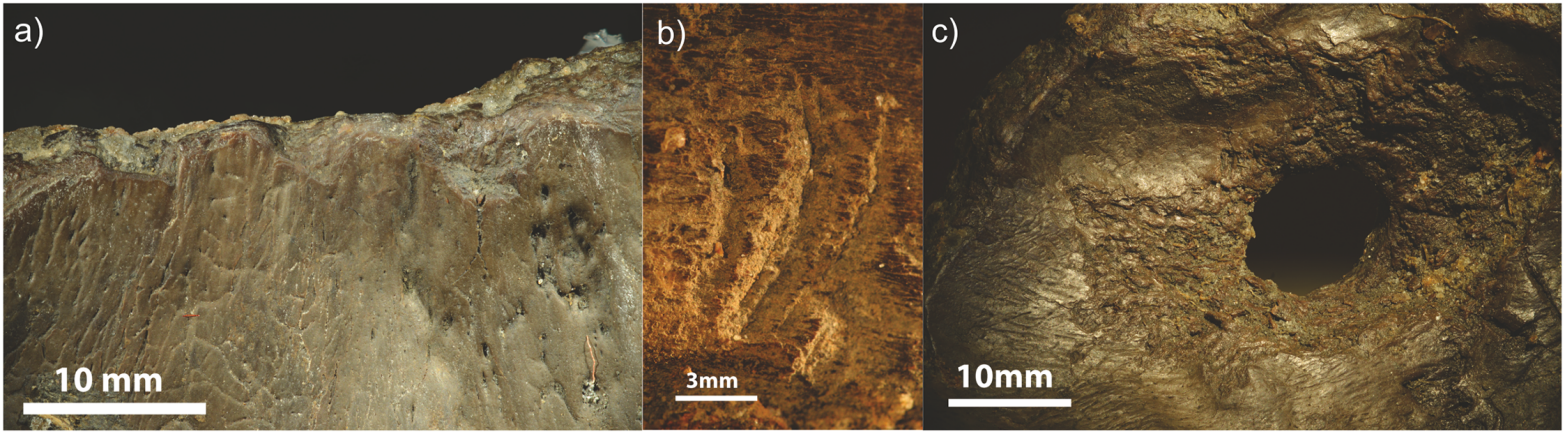
(a) Z-stack photo of scalar-shaped flakes contouring the braincase rim. (b) Z-stack photo of cut marks on the exterior of the crania, in proximity to the pedicles. (c) Z-stack photo of an ‘exploded’ appearance to the ventral side of the perforations, with linear incisions radiating out from the perforations on both sides of the skull.

Analysis revealed three areas of modification to the crania:

1. scalar-shaped flakes contouring the rim (Fig 3a)
2. cut marks on the interior and exterior of the crania, the latter in proximity to the pedicles (Fig 3b)
3. an ‘exploded’ appearance to the ventral side of the perforations (Fig 3c), with linear incisions radiating out from the perforations on both sides of the skull.

### Experimental replication

These observations prompted a series of experiments to replicate the traces visible on the headdress and thus reconstruct the process of manufacture. 103625 displays many of the classic characteristics of headdresses described by [5], including perforations, working marks around the rim and internal surfaces of the braincase, reduction of the antlers via groove-and-splinter technique and the subsequent hollowing out of the remaining beams and tines.

We used the heads of four male roe deer (*Capreolous capreolus*), four female and one male red deer (*Cervus elaphus*).

**Fig 4:**
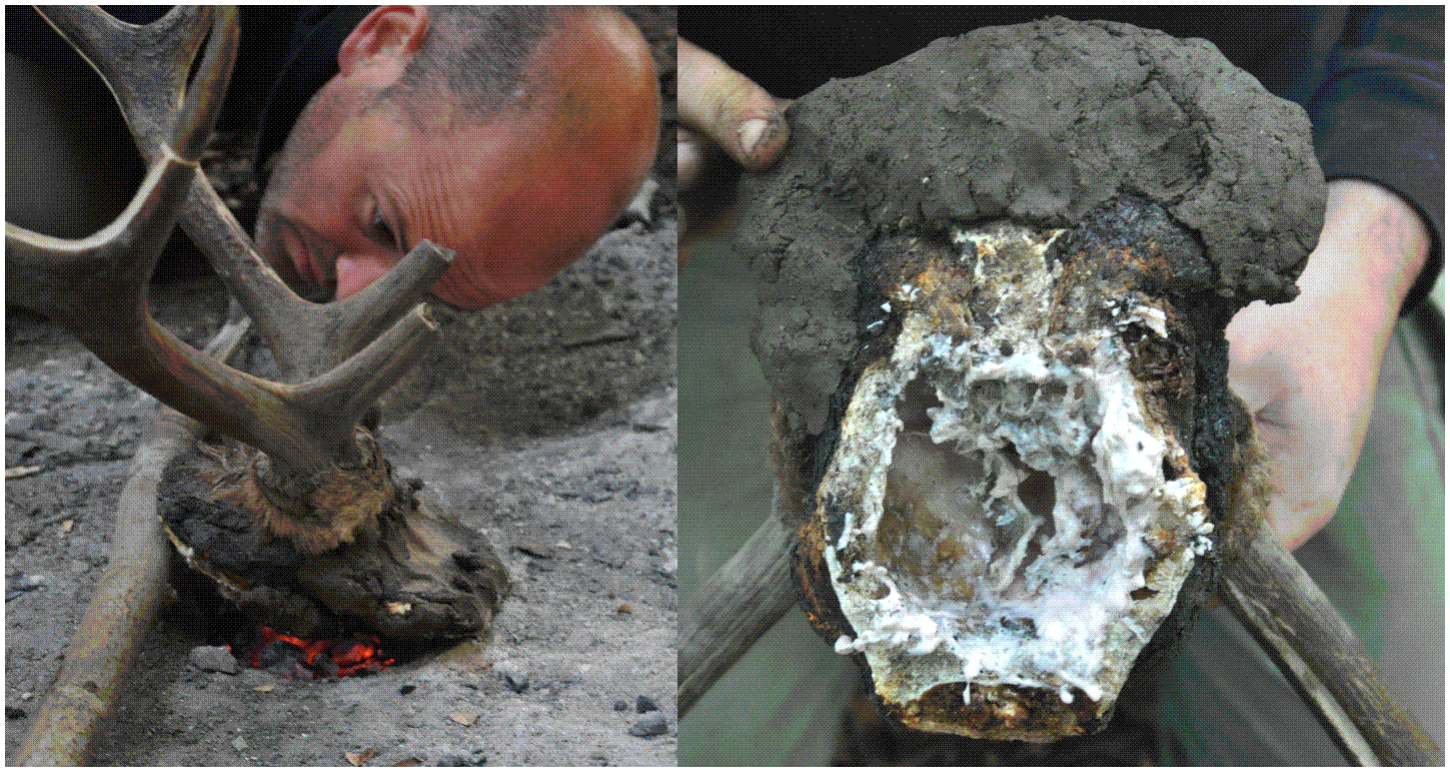
(a) placing clay-covered crania onto fire pit. (b) repacking damp clay onto burnt crania, containing cooked brain, for further firing.

*1*. We employed two different techniques to remove the upper half of the skull from the lower. The first technique involved de-skinning the head; a flint blade was then used to remove the brain from the casing and the extraneous bone. We subsequently used a large piece of flint to peck out the shape of a headdress from the crania. This method failed to produce the scalar-shaped flakes visible on the artefact. The second technique involved heat treating the lower portion of one roe deer skull and one red deer skull. A layer of damp clay was applied to the part of the head that we wanted to retain—in this case the upper part. The entire head, complete with skin, was then placed into a bed of embers for four hours. As the clay packing dried and fell off, it was replaced with fresh clay and returned to the embers (Fig 4a and 4b). Once the lower half of the crania had become charred, the base of the skull could be opened with one blow from a hammerstone.

**Fig 5:**
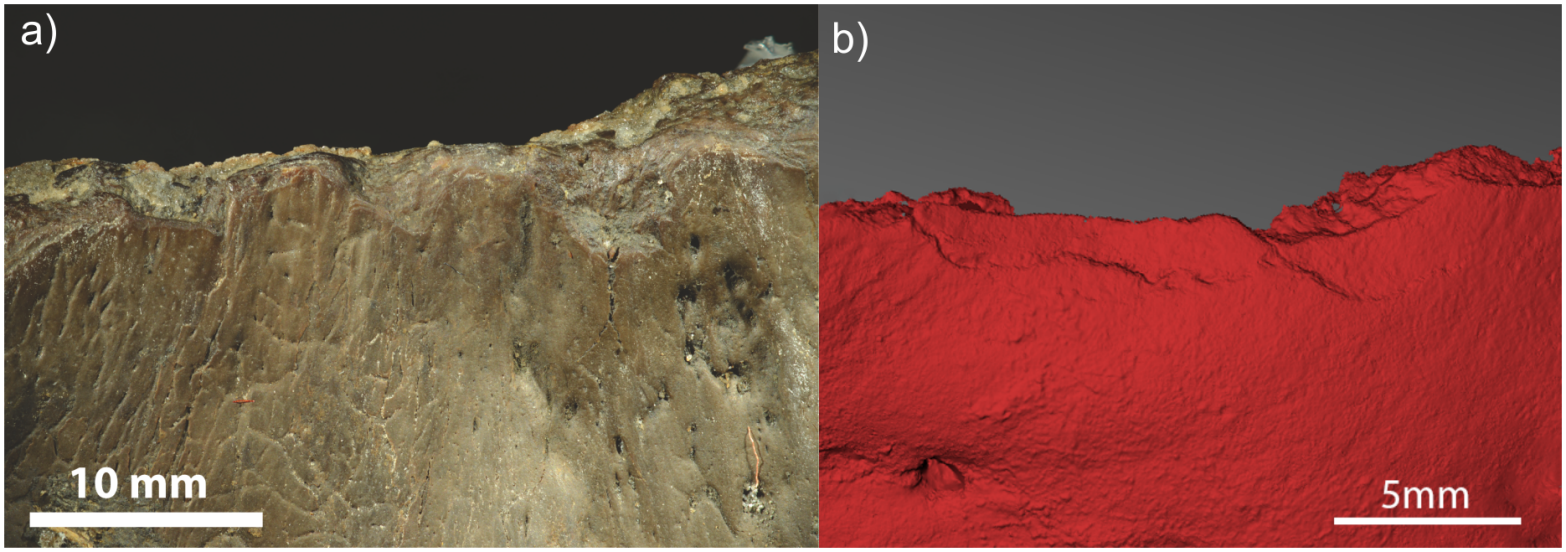
(a) Scalar-shaped flakes contouring the rim of the artefact, z-stack photo, (b) Scalar-shaped flakes contouring the rim of experimental replica, macro structured light scan.

Repetitive light percussion with a small hammerstone was used to shape the rim of the frontlet into the desired form. The skin, softened by the heat, peeled off easily. The burning was followed by repetitive blows with a hammerstone to remove the larger pieces of bone from around the base of the headdress, followed by soft blows with the flat side of the hammerstone directly onto the rim to remove smaller projecting pieces of bone. This produced the same characteristic scalar-shaped flakes evidenced along the rim of the archaeological specimen Fig 5a and 5b). The hammerstone was then used to further smooth the rim through abrasion.

**Fig 6:**
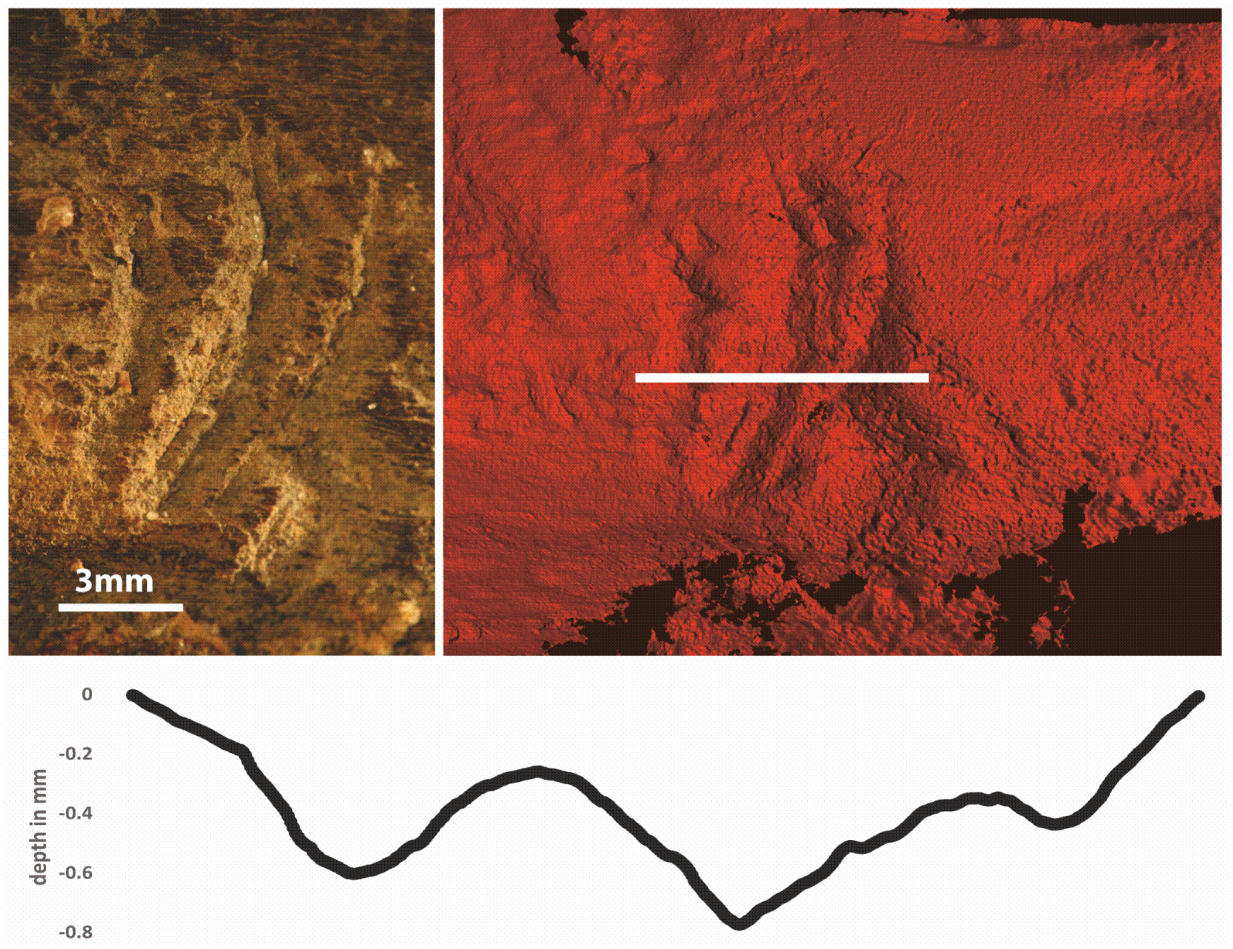
(a) Z-stack photo of features at the base of the pedicle, (b) Macro-structured light scan of the same area illustrating the location of a transect across the feature to show its profile; (c) the profile line that transects the cut-marks. The 3D characteristics of the profile show the marks were made with a chopping rather than cutting action.

*2*. When removing the brain in the method described above, we discovered that the severing of the meninges with a flint blade resulted in the creation of small cut marks on the interior of the crania. These correspond in size and shape to marks on 103625, suggesting that the brain was removed in this manner. Three parallel marks were identified on the external crania, at the base of the pedicle (Figs 3b and 6a). A profile was created by imaging the object using a high resolution macro structured light scanner, which can capture fields of view between 30 mm and 150 mm and can record a point-to-point resolution of 10 μm. Each scanned area was captured from multiple angles allowing the alignment and combination of data to create detailed surface models. The model was then digitally manipulated and perpendicular crops were taken through the feature (Fig 6b). Rather than being ‘V’ shaped, in profile these marks were oblique with a wider width-to-depth ratio, typical of a chopping action (Fig 6c), as seen at Klasies River Mouth [29]. The chopping tool may have been a flint tranchet adze; some examples recovered from Star Carr have suitable pointed terminations [4]. Our experiments established that this was a highly effective multi-functional tool for initiating de-skinning, hammering the skull into shape and for perforating the bone using the pecking method (i.e. deep transverse impacts).

What is less explicable is what activity and purpose the chop marks represent. One hypothesis is proposed: the chop marks indicate the removal of the skin. After de-skinning the head would have then been packed with clay and fired. However, as our experiments showed that removing the skin is unnecessary, this may indicate the desire to retain the deer’s hide, perhaps for it to be re-applied once the headdress had been worked into its final form and ready for wear, as seen in some ethnographic examples [24].

**Fig 7:**
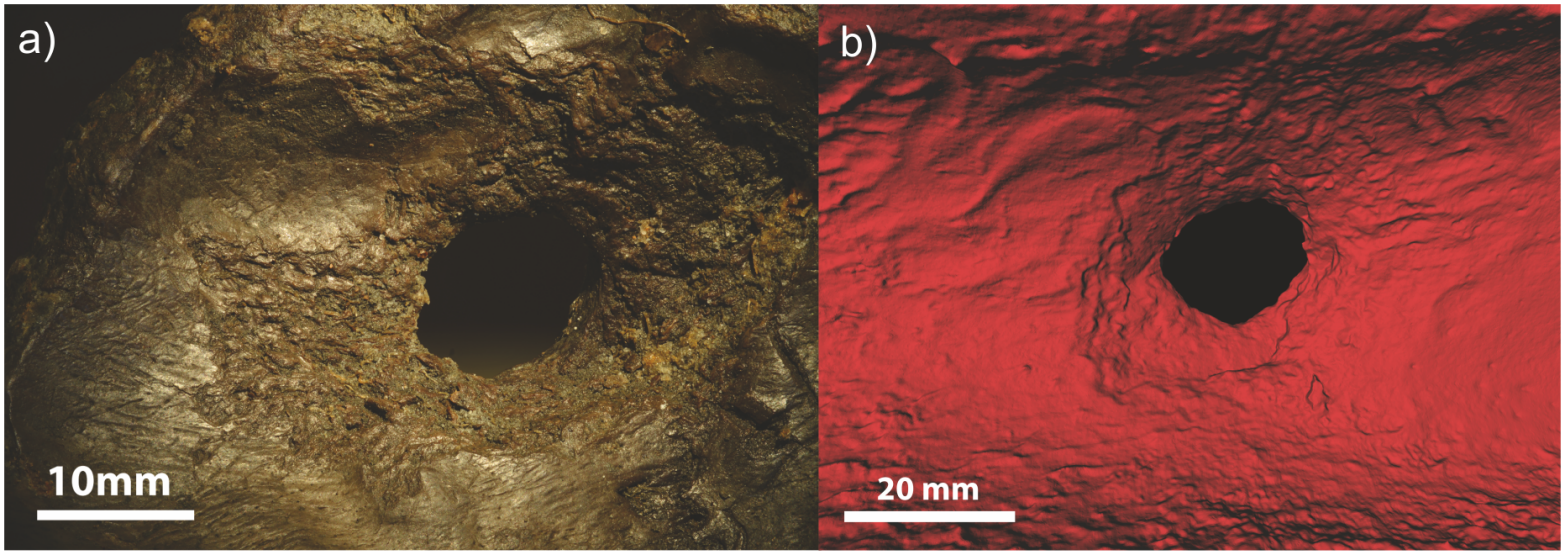
(a) Z-stack photo of ‘exploded’ appearance to the ventral side of the perforations on the artefact. (b) Replica perforation with the same ‘exploded’ appearance produced from using a core tool to peck through the crania.

*3*. Our aim was to replicate production traces visible on the headdress where two perforations have been made through the parietal bone on either side of the crania, directly below the pedicle. These displayed an ‘exploded’ appearance on the ventral side, with linear depressions extending radially from the centre of each hole. Due to the much thinner bone density of the modern red and roe deer skulls, a cow metatarsal bone was used instead. Using a core tool and the pecking technique we produced near-identical traces (Fig 7a and 7b).

It should also be noted that the headdresses found at Star Carr have had portions of the antler removed using a technique known as groove and splinter, which was used extensively at the site in the manufacture of large numbers of antler projectile points. Clark [4], the original excavator, has suggested that this was carried out in order to reduce the weight of the headdresses and make them suitable for wearing. It is a technique that would have been carefully applied, ensuring that the shape of the modified antlers retained their original form, with thin strips of the beam and brow tines intentionally left intact [15]. Experimental use of groove and splinter to work antlers, carried out by BE [30], AL and DP, suggest that this would have taken hours, possibly even days, depending on the intensity of labour invested. It is also worth noting the logistical difficulties of working antler—particularly the posterior aspect—whilst the head was attached to the body. This may suggest that the head was removed before the antlers were reduced or even that the antlers were reduced once the headdress had been used.

### Organic residue analysis

During the analysis of the frontlet organic residues (which appeared as discrete areas of sticky pale brown substance) were noted at the top of the crania, around the base of the pedicles and surrounding the fontanals. Given the aforementioned ethnographic examples for animal skins being attached to shamanic headdresses [28] we extracted ten residue samples from the surface of the crania for molecular analysis in the hope of identifying residual traces of lipids (e.g. waxes, resins) used as adhesive.

Lipids were extracted from approximately 100mg of freeze dried residues. Samples were solvent extracted using 2:1 vol/vol of DCM:MeOH (3x2 mL), sonicated for 15min. The extracts were combined, and evaporated to dryness under a stream of N_2_. Samples were silylated with BSTFA at 70°C for 1 h, and then evaporated to dryness under a gentle stream of N_2_. Derivatised samples were redissolved in *n*-hexane, and analysed directly by GC-MS using identical conditions as previously described [31–33]. This approach is commonly used to characterise resins and adhesives associated with archaeological artefacts [34]. The analysis showed that the extract contained predominantly long chain fatty acid, long chain aliphatic alcohol, phytosterol and some traces of cholesterol. As the lipid profile is consistent with the surrounding sedimentary environment it is most likely natural, with no discernible signature resulting from human activity detected.

## Results, Discussion and Conclusion

The overall manufacturing sequence of this headdress can be summarised as follows: a mature red deer male was killed in autumn or winter before the antlers were shed. The head was removed, probably superficially cleaned, before work commenced on producing the headdress. The first stage of the process may have been focused on the beams to remove a large amount of antler, some of which may have formed ‘blanks’ for the production of barbed projectile tips which were then used to hunt and fish. However, it is also possible that in some cases antler blank removal happened much later after the headdress had been used; in which case the process functioned either as a form of decommissioning of the headdress and/or the recycling of antler. Given the amount of worked antler present at the site, including over 200 barbed projectile tips, this latter theory is not implausible.

The skull itself was first worked in an *ad hoc* manner with a core tool. Emphasis was placed on achieving a desired form rather than labouring over refinement of edges/surfaces.

At this point there are two diverging hypotheses: a tool was used to chop through the skin, initiating the de-skinning process; *or* the skin was left on, the upper half of the cranium covered with damp clay, before being placed into the embers of a fire. The skull was subsequently retrieved, and the charred bone removed using a small hammerstone. After removing the clay, the skin (if remaining) was peeled away from the frontal and parietal bones. The cranium was defleshed with muscle attachments below the pedicle being chopped with a core tool, and the brain, which experiments show was cooked to perfection for consumption with this method, then removed with a flint blade. Perforations on each side of the cranium were made using a hand-held flint core tool (possibly the same pointed tranchet adze used for initiating de-skinning and detaching muscle).

Our work raises new questions regarding the precise form of the headdress when worn in a shamanic context. It has been impossible to determine the exact point at which the antlers were reduced into the shape observed on the recovered artefacts. If reduction occurred prior to the modification of the cranial bones it can be variously argued that this was a functional solution to minimising the total weight of the object, a way of controlling and defining the intended form of the headdress, as well as providing raw material which could be used to create other forms of antler artefacts such as projectile points. It is, however, possible that the other factors influenced the decision to reduce the antlers in the manner observed on headdress 103625. Evenki shaman are known to enhance the sharpness of antlers in order to make them more ‘spear-like’ as they take soul-flights to negotiate, and if need be, fight with spirits encountered on different levels of the sky when in a trance state [28]. In either case, if the antlers were reduced post-cranial modification, and potentially post-use, then this implies that the antlers were intact when the headdress was worn. In this scenario, the form of excavated examples is not as representative of the shaman’s headdress as previously believed, but instead represents later recycling of antler. In the case of headdress 103625, antler was removed in a way which allowed both for the creation of other antler objects, and the retention of specific aspects of the antlers’ original form. The possible recycling of raw material for tool production from a shaman’s headdress would suggest a strong physical and symbolic act of decommissioning was at play.

The analysis presented here represents the first scientific study of the oldest known evidence of a shamanic costume, and as such forms a major contribution to the understanding of some of the earliest religious artefacts in the world. We have demonstrated the techniques and methods used by hunter-gatherers to transform red deer heads into bone and antler headdresses 11 000 years ago in North Western Europe. Whilst new questions arise from this work, this research challenges previously held assumptions over the care and time invested in the modification of the animal’s “skull cap” in order to create these ritualistic artefacts, and instead suggests that this was achieved through expedient manufacturing techniques that incorporated the use of pyrotechnology.
